# P-682. Rates of averted pediatric pneumonia episodes following PCV implementation vary significantly according to age, population characteristics and clinical status

**DOI:** 10.1093/ofid/ofaf695.895

**Published:** 2026-01-11

**Authors:** Ron Dagan, David Greenberg, Guy Hazan, Bart A van der Beek

**Affiliations:** Ben-Gurion University of the Negev, Beer Sheva, HaDarom, Israel; Soroka University Medical Center, Pediatric Infectious Disease Unit, Beer Sheva, HaDarom, Israel; Soroka University Medical Center, Pediatric Department D, Beer Sheva, HaDarom, Israel; Ben Gurion University, Faculty of Health Sciences, Beer Sheva, HaDarom, Israel

## Abstract

**Background:**

A global decline in pediatric community-acquired alveolar pneumonia (CAAP) followed PCV implementation. We used 15 years of population-based active surveillance on pediatric CAAP to assess the impact of age, population, and clinical status on averted episodes.

**Figure:**
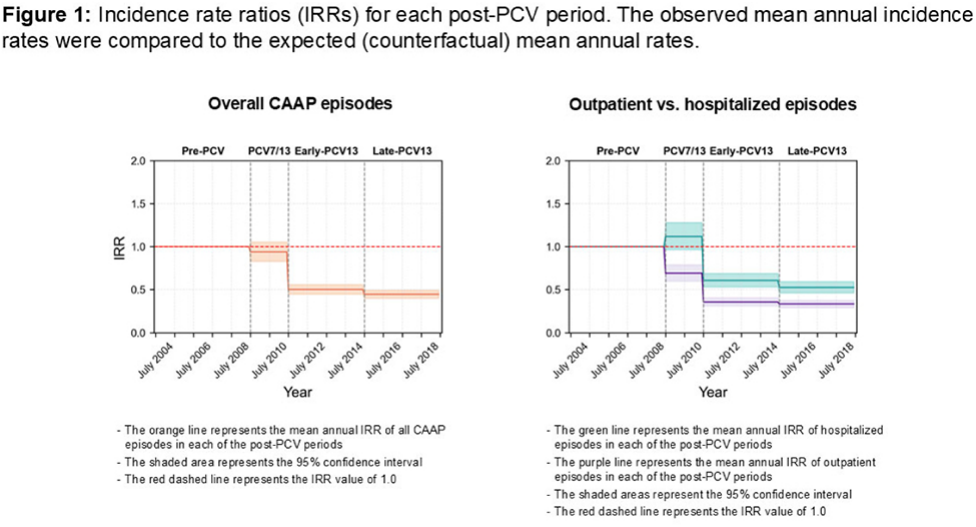

**Figure:**
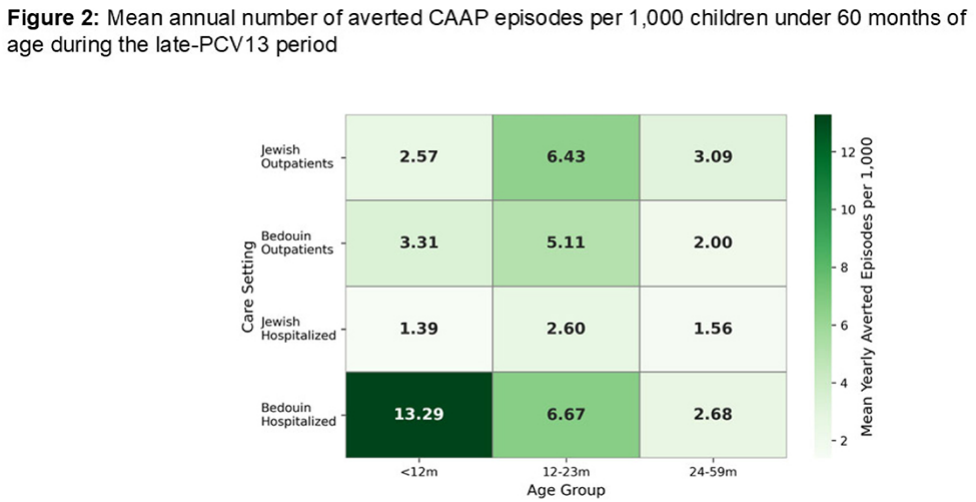

**Methods:**

This is an ongoing, population-based active surveillance of CAAP-related hospital utilization in children < 60 months, initiated in 2004 (Ben-Shimol, CID, 2020;71;1770). All CAAP-related hospital visits in southern Israel were included. CAAP was radiographically confirmed by consensus reading. Hospital visits were classified as hospitalized or outpatient. A negative binomial model based on monthly cases assessed PCV13 impact. Two populations reside in our region: Jewish (high/middle SES) and Bedouins (low/middle SES). Epidemiologic years ran July-June. Study periods: Pre-PCV (2004–09), PCV7/13 (2009–11), Early PCV13 (2011–15), Late PCV13 (2015–19). Mean annual averted episodes were determined by subtracting the observed mean annual incidence rates from the expected rates (and 95% CI) during late PCV13.

**Figure:**
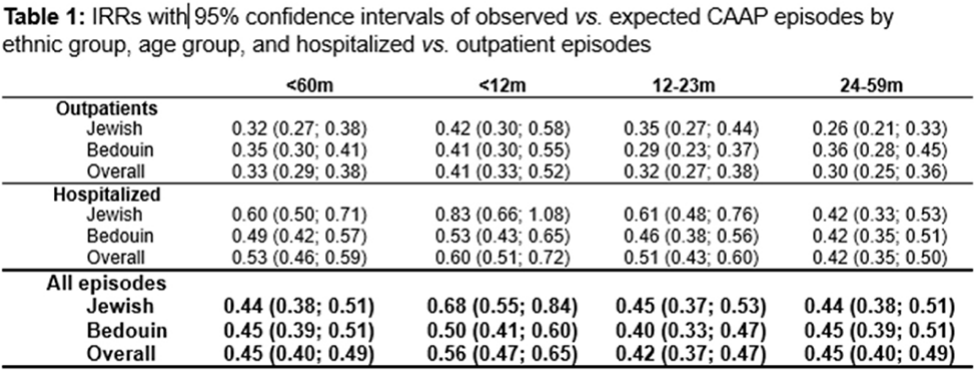

**Figure:**
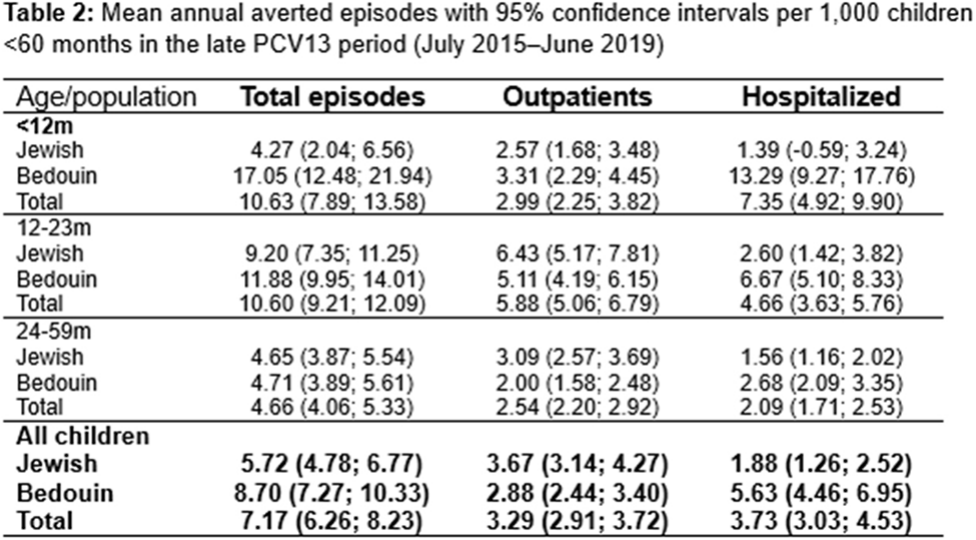

**Results:**

We studied 11,310 episodes (3,677 outpatient; 7,633 hospitalizations). During the late PCV13 period, rate reduction was significantly greater in outpatients (67%; 95% CI 62-71%) *vs*. inpatients (47%; 95% CI 41-54%)) (Figure 1, Table 1). Similar patterns were seen across ages and both populations. However, baseline hospitalized and outpatient rates differed by age and population, leading to marked differences in averted episode rates (Table 2, Figure 2) The largest mean annual reduction/1,000 was in hospitalized Bedouins < 12m (13.29 *vs.* 1.39 in Jews; 9.6-fold), followed by Bedouins 12–23m (6.67 vs 2.60; 2.6-fold). Overall averted hospital visits for CAAP spanning the first 5 years of life were ∼4.4% and 2.9% of the Bedouin and Jewish children, respectively.

**Conclusion:**

PCV implementation in Israel, averted a large number of hospital visits for outpatient and hospitalized CAAP episodes in early childhood. Impact was greatest among the most vulnerable population and age groups, constituting an important step toward equity by vaccination.

**Disclosures:**

Ron Dagan, Professor MD, GSK: Advisor/Consultant|GSK: Honoraria|Medimmune/AstraZeneca: Grant/Research Support|Medimmune/AstraZeneca: Honoraria|MSD: Advisor/Consultant|MSD: Grant/Research Support|MSD: Honoraria|Pfizer: Advisor/Consultant|Pfizer: Grant/Research Support|Pfizer: Honoraria|Sanofi pasteur: Honoraria David Greenberg, Professor MD, GSK: Advisor/Consultant|GSK: Honoraria|MSD: Advisor/Consultant|MSD: Grant/Research Support|MSD: Honoraria|Pfizer: Advisor/Consultant|Pfizer: Honoraria

